# Delayed Diagnosis of Infected Posterior Mediastinal Teratoma

**Published:** 2013-06-22

**Authors:** KR Bharath Kumar Reddy, G Rajashekhar Murthy, Sanjay KS

**Affiliations:** Department of Pediatrics, Indira Gandhi Institute of Child Health, Bangalore

**Keywords:** Infected teratoma, Mediastinum, Empyema thoracic

## Abstract

Mediastinal teratomas are rarely reported in children. We report a 12-year-old child, diagnosed and treated as empyema thoracis in a peripheral setting for 18 months. In our centre, CT scan performed revealed a well circumscribed multiloculated cystic lesion containing fat, bone and teeth, suggestive of a teratoma. The mass was excised and found to be an infected posterior mediastinal teratoma, confirmed on histopathology.

## INTRODUCTION

The most common sites of teratomas in children include sacrococcygeal (40%), ovary (25%), testicle (12%), brain (5%), and rarely other areas including the neck and mediastinum (18%) [1]. Mediastinal teratomas are found to be asymptomatic in more than half the patients and discovered on radiological evaluation [1]. Though many are asymptomatic, these tumors need to be diagnosed early and resected to prevent further morbidity and complications either due to compression effects, rupture of the tumor or secretion of hormones and enzymes. These tumors can masquerade as a multiloculated empyema on plain radiography. In the absence of chest CT scan, these tumors can be missed and appropriate treatment can significantly be delayed. Herein, we report a case of infected posterior mediastinal teratoma which was misdiagnosed as empyema and treated for the same.

## CASE REPORT

A 12-year-old boy presented with complaints of fever and cough occurring on and off for 2 years. The child was diagnosed as empyema by the local physician and an intercostal drain was inserted which drained minimal foul smelling fluid. The drain was apparently retained for 3 months in view of persistent discharge and child was treated with anti-tubercular therapy for 9 months. For last 6 months, he complained of progressive dyspnoea, not associated with any postural variations. He had a sinus in the chest which was persistently draining 5-10 ml of foul smelling fluid daily. He had a history of significant weight loss and decreased appetite for 6 months. There was no history of contact with tuberculosis in the family. On examination he was found to be wasted with severe acute malnutrition. General physical examination revealed severe pallor with grade III clubbing. Respiratory system examination revealed a bulging chest on the right side with decreased movements. The same areas were found to be dull on percussion. Bronchial breathing was present in the right infraclavicular and interscapular regions. All the other systems were within normal limits. Chest X ray (Fig. 1) showed a homogenous opacity on the right side with a fluid level, with clear costophrenic angles. Calcification was noted in the right 6th intercostal spaces with crowding of ribs in the upper zones. USG chest showed evidence of minimal (1.4mm) loculated collection in right posterior basal region. CT chest (Fig. 2) done revealed a well circumscribed peripherally enhancing multiloculated cystic fluid and a soft tissue density lesion with fat components, well defined multiple calcified tooth like structures and bone pieces. There were associated patchy sub-segmental non homogenous opacities in apical and posterior segment of right upper lobe. Multiple small non-calcified, non-necrotic right paratracheal, pre-tracheal and subcarinal lymphadenopathy were noted. The cultures from the sinus tract grew pseudomonas aeruginosa and serratia marcescens sensitive to ceftazidime and amikacin. The patient thus received a course of antibiotics for 2 weeks. The fever subsequently subsided. Serum alpha protein levels were within normal limits.

A right sided thoracotomy was done and tumour was excised en-bloc. A white-gray colored, well circumscribed, and thick capsulated mass was removed. No communication between the bronchi and the mass were noted. The teratoma was found to be originating from the posterior mediastinum (Fig. 3). A tube thoracostomy was left, the collapsed right lung was re-expanded, and the patient was extubated. Postoperative course remained uneventful. Gross pathological examination showed a 10x6x3 cm mass with 2 skin covered nodules with grey yellow (fatty) and bony hard areas within focally cystic areas on cut section. Histopathological examination showed areas of fat, bone, teeth and lung tissue with normal skin tissue on the covering and with occasional neural tissue present, suggestive of a mature teratoma.

**Figure:**
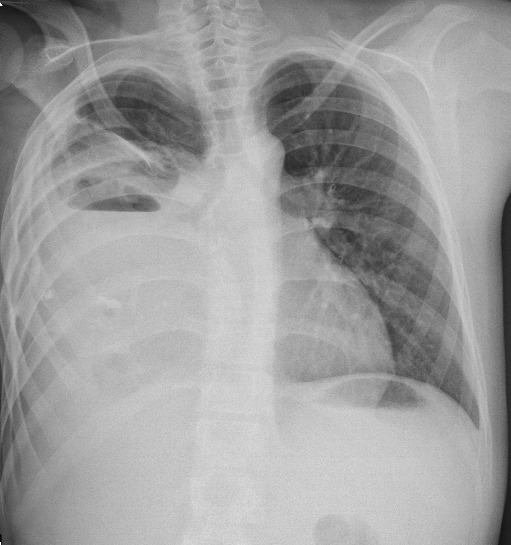
Figure 1: Chest X-ray showing a homogenous opacity in the right hemithorax with scattered calcification.

**Figure:**
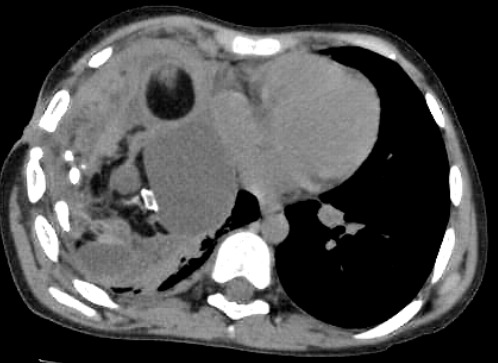
Figure 2: CT scan of thorax shows calcified lesions in a cyst in the mediastinum suggestive of bone and teeth in the mass.

**Figure:**
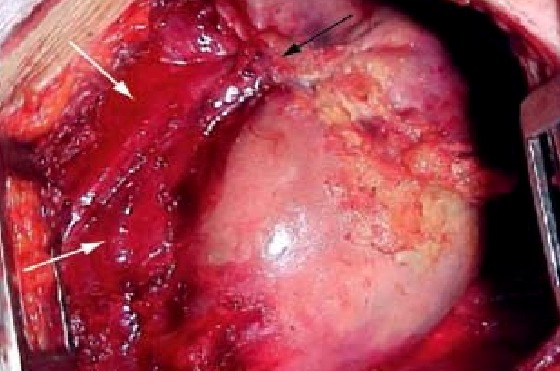
Figure 3: Intraoperative finding of a well circumscribed posterior mediastinal mass compressing on the adjacent lung tissue.

## DISCUSSION

Of the mediastinal teratomas, most are seen to arise from the anterior mediastinum with 2-8% from the posterior mediastinum [2, 3]. As mediastinal teratomas can present with symptoms such as cough, dyspnea and chest pain, they can be misdiagnosed as empyema especially on plain radiography. On radiography, calcifications are seen in 26% of patients. The chest X-ray of our patient does show calcification which could have been a marker to favor a teratoma in comparison to an empyema. A lateral chest X-ray could have helped to aid the diagnosis in our child [4]. The presence of calcifications on a chest X-ray is an indication for more advanced imaging such as a CT scan in this patient. We do note that if a CT scan had been done in our patient at an earlier stage by the primary care pediatrician, the tumor would have definitely been picked up earlier; appropriate treatment instituted immediately and thus reduced complications in him. 

Complete resection of the tumor is the treatment of choice. Clinical improvement was noted in our patient following surgical excision of the mass. A similar case of an infected mature teratoma of the mediastinum having been missed was reported highlighting the need for more awareness of this entity among pediatricians in developing countries [5]. The delay in subjecting the patient to advanced and modern day imaging which led to the misdiagnosis reflects on the lack of adequate training in pediatric surgery and oncology among primary care physicians in our part of the world. The patient’s significant malnutrition, sinus tract formation, recurrent infections and exposure to drug toxicities could have been avoided by an appropriate diagnosis. Thus, this case report highlights the need for early advance imaging such as a CT scan or MRI of the thorax in children not responding to routine treatment of an empyema. At the level of a primary care physician, we would suggest that a child be referred for either advanced imaging or a pediatric surgery opinion in cases which do not respond to treatment within the expected time. The delay in parental seeking of specialist care for the same can be attributed to illiteracy, lower socioeconomic status and cultural issues associated with going to an advanced center. Thus, the onus of diagnosis, early and appropriate investigations and referral to a specialist lies in the hands of the treating primary care physician.

## Footnotes

**Source of Support:** Nil

**Conflict of Interest:** None declared

